# Neuronal migration defects in the Loa dynein mutant mouse

**DOI:** 10.1186/1749-8104-6-26

**Published:** 2011-05-25

**Authors:** Kassandra M Ori-McKenney, Richard B Vallee

**Affiliations:** 1 Department of Pathology and Cell Biology, Columbia University, New York, NY 10032, USA; 2 Department of Physiology, University of California, San Francisco, San Francisco, CA 94158, USA

## Abstract

**Background:**

Cytoplasmic dynein and its regulatory proteins have been implicated in neuronal and non-neuronal cell migration. A genetic model for analyzing the role of cytoplasmic dynein specifically in these processes has, however, been lacking. The Loa (Legs at odd angles) mouse with a mutation in the dynein heavy chain has been the focus of an increasing number of studies for its role in neuron degeneration. Despite the location of this mutation in the tail domain of the dynein heavy chain, we previously found a striking effect on coordination between the two dynein motor domains, resulting in a defect in dynein run length *in vitro *and *in vivo*.

**Results:**

We have now tested for effects of the Loa mutation on neuronal migration in the developing neocortex. Loa homozygotes showed clear defects in neocortical lamination and neuronal migration resulting from a reduction in the rate of radial migration of bipolar neurons.

**Conclusions:**

These results present a new genetic model for understanding the dynein pathway and its functions during neuronal migration. They also provide the first evidence for a link between dynein processivity and somal movement, which is essential for proper development of the brain.

## Background

Cytoplasmic dynein is a minus-end directed microtubule motor protein involved in a wide variety of functions. In order to perform such diverse activities in the cell, dynein makes use of numerous regulatory partners, including dynactin, LIS1, NudE and NudEL [1-3]. LIS1 and NudE/L, in particular, have received considerable attention for their role in brain development [4-7]. Sporadic mutations in LIS1 cause type I, or classical, lissencephaly, a severe brain developmental disease characterized by a smooth cortical surface [8]. Patients with lissencephaly exhibit lamination defects of the cerebral cortex, consistent with a defect in neuronal migration. Developmental analysis of LIS1 mutant mouse lines has shown that a reduction of LIS1 protein leads to neocortical organization defects in a dose-dependent manner [5,9-11]. Live analysis of embryonic rat brain subjected to LIS1 RNA interference (RNAi) has directly demonstrated defects in neural progenitor cell division, radial migration, and axon elongation [7,12]. The genes encoding NudE and NudEL, Nde1 and Ndel1, are closely related and interact genetically with LIS1 and cytoplasmic dynein [13]. Nde1 and Ndel1 mutant mice exhibit either microcephalic or lissencephalic brain phenotypes, consistent with a functional relationship to LIS1 [6,14-16].

Several studies have focused on the role of genes in the dynein pathway in mammalian brain development. However, there are no genetic models with which to study the involvement of dynein directly. Mice homozygous null for the cytoplasmic dynein heavy chain gene exhibit early embryonic lethality, whereas heterozygotes show no obvious abnormalities [17]. However, several dynein mutant mouse strains have since been identified in screens for genes involved in neurodegeneration [18,19]. Of these dynein mutants, the Loa mutation has received particular attention [20,21]. The Loa heterozygous mice are viable and develop early onset neurodegenerative disease, but the homozygous animals die perinatally due to an inability to feed [18]. Interestingly, both the Loa/+ and the Loa/Loa mice exhibited defects in retrograde axonal transport [18,22]. The Loa mutation resides in the amino-terminal tail region of the dynein heavy chain. Nonetheless, enzymatic, biochemical, and single molecule analysis revealed a decreased microtubule affinity for the Loa mutant dynein due to a disruption in the coordination of its two motor domains [23]. This defect was manifested as a decrease in processivity for purified mutant dynein in *in vitro *single molecule assays. Particle tracking of lysosome/late endosome transport in cultured wild-type and Loa hippocampal neurons revealed a marked reduction in vesicular run lengths that could be accounted for quantitatively by the reduced processivity of the mutant dynein [23].

The Loa mouse represents an important model for the study of neurodegeneration. It should also, in principle, be useful in evaluating the role of dynein in other functions, such as neuronal migration. In view of its particular effects on dynein mechanochemical behavior, Loa also affords an opportunity to test whether dynein processivity contributes to this process. The Loa/Loa mice have been reported to display abnormal facial motor neuron organization, suggesting potential cellular migration defects [18]. We report direct evidence that the Loa mutation disrupts this process, causing a delay in neocortical development, a result that confirms a more general value for this mutant mouse strain and has implications for the role of motor processivity in neuronal migration.

## Results

### Lamination defects in homozygous Loa mutant mice

**Figure 1:**
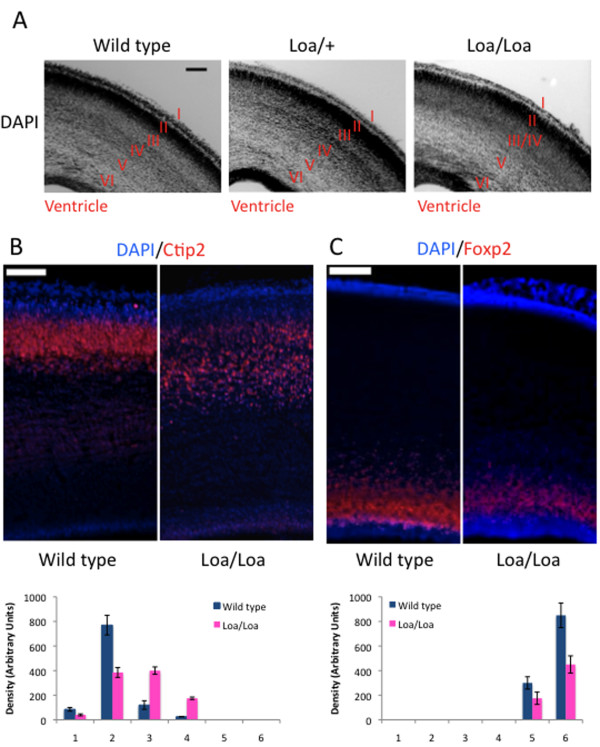
**Cortical defects are apparent in the brains of Loa/Loa animals, but not in the brains of Loa/+ or wild-type mice**. **(A) **Brain sections stained with DAPI reveal six distinct layers, labeled I to VI, in wild-type and Loa/+ brains, but blurred layer boundaries in the Loa/Loa brains. Scale bar = 200 μm. **(B) **Wild-type and Loa/Loa brain slices stained with Ctip2, which labels neurons in layers II/III. Brain sections were divided into six equal regions and the corresponding graph shows the mean Ctip2 density within each of these regions for each genotype. Error bars represent the standard deviation. Scale bar = 200 μm. **(C) **Wild-type and Loa/Loa brain slices stained with Foxp2, which labels neurons in layer VI. Brain sections were divided into six equal regions and the corresponding graph shows the mean Foxp2 density within each of these regions for each genotype. Error bars represent the standard deviation. Scale bar = 200 μm.

To test whether the Loa mutation affects brain development, we first examined the lamination pattern of the cerebral cortex in postnatal day zero (P0) mouse pups. Wild-type and Loa/Loa brains had a similar cortical thickness: 1,198 ± 150 μm for wild type versus 1,220 ± 160 μm for Loa/Loa (*P *= 0.8705; n = 3 brains per genotype). Six distinct layers could be identified by DAPI staining in both wild-type and Loa/+ brains; however, the boundaries were blurred in the Loa/Loa brains, especially between neuronal layers III and IV (Figure 1A). To identify individual layers, we measured the density of Ctip2 and Foxp2 staining, which serve as markers for layers III and VI, respectively. In the Loa/Loa brains, Ctip2-positive cells were spread over an area roughly twice as broad as the compact layer observed in the wild-type brain (Figure 1B; *P *< 0.005; n = 3 brains per genotype). In contrast, there were fewer Foxp2-positive cells in layer VI of the Loa/Loa brain compared with wild type (Figure 1C; *P *< 0.005; n = 3 brains per genotype), a further indication of cortical disorganization.

**Figure 2:**
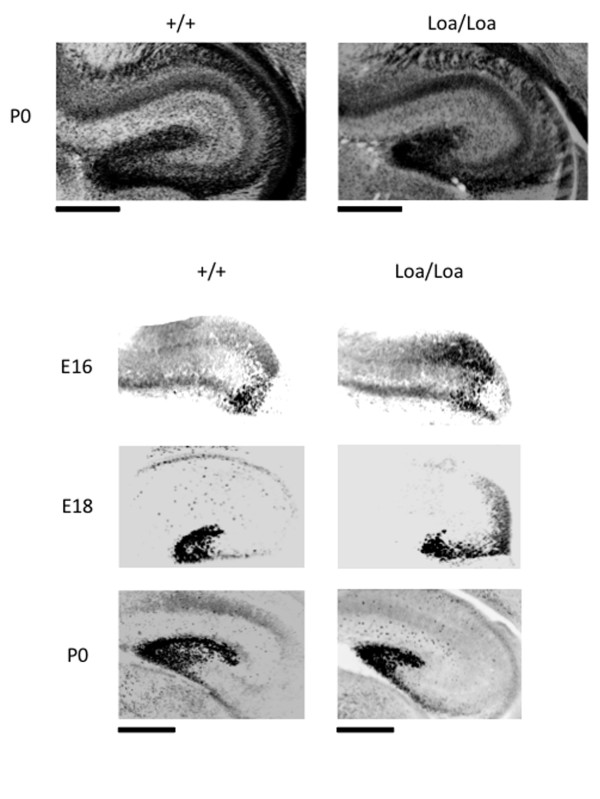
**Loa mutant mouse brains exhibit a delay in hippocampal radial migration**. **(A) **P0 brain slices stained with DAPI reveal a normal hippocampal structure, but a smaller dentate gyrus in the Loa/Loa compared with wild-type brains. **(B) **Embryonic day (E) 16, E18 and P0 brains were stained with Prox-1, a marker for granule neurons that migrate to form the dentate gyrus of the hippocampus. The progression of Prox-1-labeled neurons is delayed in the Loa/Loa brain compared with the wild-type brain. Scale bars = 200 μm.

We also examined the hippocampus of the Loa/Loa mutant brain and noticed that, although overall structure was normal, the dentate gyrus was smaller than in the wild-type brain (Figure 2A). We therefore analyzed the subset of granule neurons (labeled by Prox-1) that are born in the subventricular zone of the neocortex and migrate to form the dentate gyrus [24]. In the Loa/Loa hippocampus, granule cell redistribution to the dentate gyrus was delayed at all time-points examined, and by P0 the dentate gyrus was smaller and more compact than in the wild-type hippocampus (Figure 2B). Together our data revealed lamination defects in both the neocortical and hippocampal regions of the Loa/Loa mouse that were similar to, though less severe than, those reported in the LIS1 compound heterozygous mutant mice [9,10].

### Loa/Loa brains exhibit defects in neuronal migration

**Figure 3:**
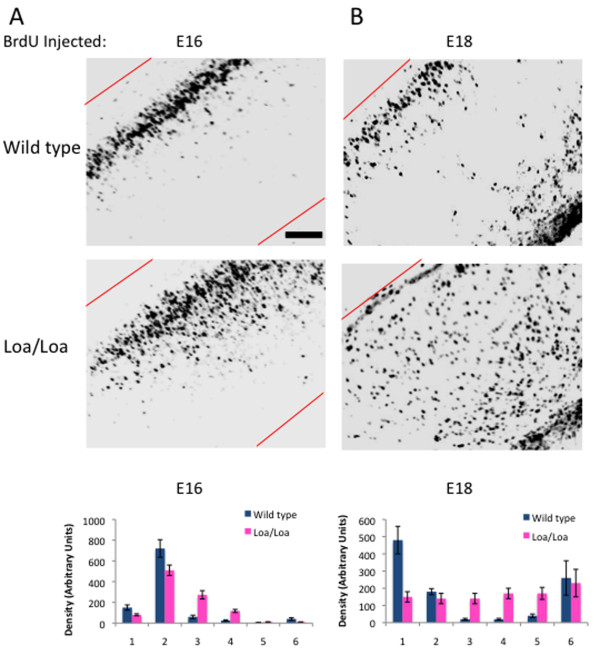
**BrdU birthdating study reveals defects in neuronal migration in the Loa/Loa mutants**. **(A,B) **Pregnant mice were injected with BrdU at either E16 (A) or E18 (B), and wild-type and Loa/Loa littermates were sacrificed at P0. (A) Migration of E16 BrdU-labeled neurons in wild-type and Loa/Loa brain slices reveals a delay in migration for the Loa/Loa neurons, which is quantified in the graph below (mean density of BrdU-positive cells ± standard deviation). (B) Migration of E18 BrdU-labeled neurons in wild-type and Loa/Loa brain slices reveals a delay in migration for the Loa/Loa neurons, which is quantified in the graph below (mean density of BrdU-positive cells ± standard deviation). Scale bar = 200 μm.

To explore the origin of the lamination abnormalities, we first performed a BrdU birthdating study by injection of pregnant Loa/+ mice with the thymidine analog 5-bromo-2'-deoxyuridine (BrdU) at either embryonic day (E) 16 or 18. BrdU is incorporated into the DNA of S-phase progenitor cells and serves as a stable marker for cells born around the time of injection. Brains from pups exposed to the label at E16 or E18 were analyzed for the distribution of BrdU-positive cells at P0. In wild-type brains, E16 BrdU-labeled cells formed a compact layer near the pial surface by P0 (Figure 3A). In the Loa/Loa brains, the majority of BrdU-positive cells migrated to the same cortical region of the brain, but formed a broader layer and showed many trailing neurons (Figure 3A; *P *< 0.005 for cell density within the first two cortical layers; n = 3 brains per genotype). A similar effect was observed with neurons labeled with BrdU at E18: the neurons showed a reduced migration distance, as well as a more scattered distribution in the Loa/Loa mutants relative to wild-type controls (Figure 3B; *P *< 0.001 for cell density within the first two cortical layers; n = 3 brains per genotype).

**Figure 4:**
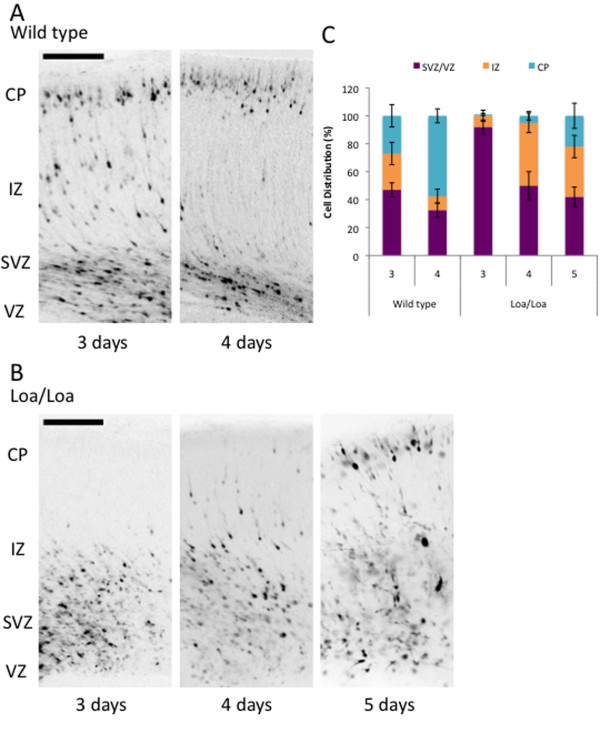
***In utero *electroporation assay exposes a two-day migratory delay in the Loa/Loa mice**. **(A,B) **Coronal sections of a wild-type (A) or Loa/Loa (B) mouse brain 3, 4, or 5 days after electroporation at E15 with GFP cDNA. In the wild-type brain (A), GFP-positive neurons migrated radially from the ventricular zone (VZ) to cortical plate (CP) with increasing time, while in the Loa/Loa brain (B), GFP-positive neurons migrated more slowly and did not reach the CP until day 5. Scale bar = 100 μm. **(C) **Percentage of GFP-positive cells in different regions of the neocortex in wild-type and Loa/Loa brains. Error bars represent the standard deviation. At least three mutant brains and three littermate control brains were analyzed at each day after electroporation. CP, cortical plate; IZ, intermediate zone; SVZ, subventricular zone; VZ, ventricular zone.

We also introduced GFP cDNA into neural progenitor cells in the mouse neocortex by *in utero *electroporation at E15 [25]. GFP-positive cells in the ventricular zone/subventricular zone, intermediate zone (IZ), and cortical plate (CP) were examined 3, 4, or 5 days later (Figure 4; n = 3 brains per genotype, per time-point). In the wild-type brains, 27% of the transfected neurons had reached the CP by day 3 (E18), and 57.5% by day 4 (Figure 4A,C), a time-course consistent with previous observations [26,27]. In Loa/Loa brains, however, the majority of transfected cells remained within the ventricular zone and subventricular zone at both day 3 and day 4, with only a subset having reached the IZ (Figure 4B,C; 45% by day 4; *P *< 0.01). However, by day 5, the distribution of GFP-positive cells in Loa/Loa brains was almost identical to that at day 3 in the wild-type brains (Figure 4B,C; 22% in the CP of Loa/Loa brains). Thus, the Loa mutation delays the radial redistribution to the CP by approximately 2 days in the mouse neocortex.

### Live cell imaging reveals a decreased rate of somal translocation

**Figure 5:**
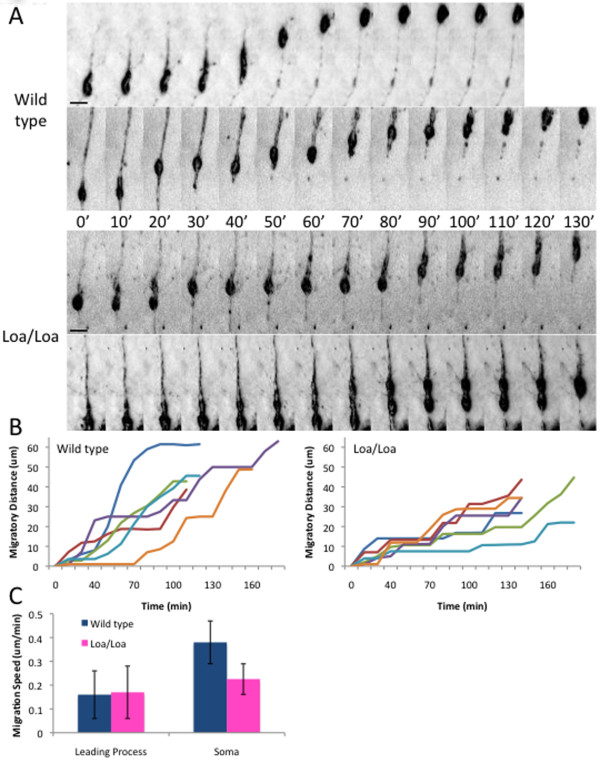
**Live cell imaging of radial migration in the intermediate zone**. Mouse brains were sectioned and incubated with Oregon Green BAPTA-1 488 AM, and the labeled bipolar cells were imaged within the IZ. **(A) **Kymographs of somal translocation within the neurons of the wild-type and Loa/Loa mouse brains. Time is shown in minutes. Scale bar = 10 μm. **(B) **Tracings of cell body position for six representative wild-type neurons and six representative Loa/Loa neurons show a slower progression of somal movement in the Loa/Loa neurons. **(C) **Graph depicting the average rates of leading process outgrowth and somal translocation (± standard deviation) for wild-type and Loa/Loa neurons (*P *< 0.0001 for somal translocation; n = 12 cells from at least three brains per genotype followed for at least 110 minutes).

To obtain further insight into the basis for the delay in neuronal redistribution, we performed live cell imaging of E16 wild-type and Loa/Loa brain slices labeled with Oregon Green BAPTA AM. Labeled cells within the IZ exhibited the normal bipolar morphology of radially migrating neurons. The rates of neuronal movement towards the pial surface varied with time and from cell to cell as previously reported [12,28,29] (Figure 5). The tip of the leading process progressed at a similar average rate for both wild-type and Loa/Loa neurons (0.16 ± 0.10 μm/minute and 0.17 ± 0.11 μm/minute, respectively; *P *= 0.817). However, the rate of somal translocation was significantly different between genotypes, with an average rate of 0.38 ± .09 μm/minute for wild-type neurons, but only 0.23 ± 0.6 μm/minute for Loa/Loa neurons (Figure 5A-C; *P *< 0.0001; n = 12 cells from at least three brains per genotype followed for at least 110 minutes).

Many cell bodies of the migrating wild-type and Loa/Loa neurons were distorted into an elongated shape during migration. However, the wild-type cell bodies remained in this stretched conformation for 20.0 ± 11.5 minutes before returning to their normal rounded morphology after advancing, whereas Loa/Loa cell bodies remained in the stretched state for 38.0 ± 13.0 minutes (Figure 5A,B; *P *< 0.05; n = 12). These results are indicative of a prolonged somal translocation state due to the reduced transport by the Loa mutant motors.

### Changes in mitotic progression are not detected in Loa progenitors

**Figure 6:**
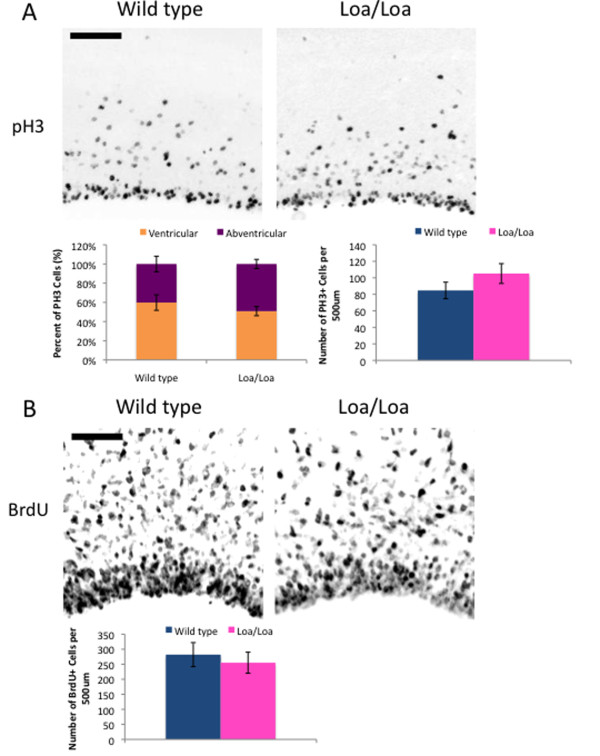
**Mitosis is not significantly affected in the Loa/Loa brains**. **(A) **Wild-type and Loa/Loa brain sections immunostained with PH3, which is expressed during mitosis. There was no significant difference in the location of mitotic cells (either in ventricular or abventricular regions), or in the number of mitotic cells between the two genotypes, as depicted in the corresponding graphs (average number of cells ± standard deviation). Scale bar = 100 μm. **(B) **BrdU pulse-labeling of wild-type and Loa/Loa embryos reveals no significant difference between the number of BrdU-labeled cells as shown in the images and graph (average number of cells ± standard deviation). Scale bar = 100 μm.

Cytoplasmic dynein is involved in numerous aspects of mitosis, including mitotic spindle organization and orientation, interactions of kinetochores with microtubules, and mitotic checkpoint regulation [30-34]. To test for accumulation of dynein mutant cells in mitosis, we stained wild-type and Loa/Loa E16 brain slices with an antibody to phospho-histone 3 (PH3). Although we did find a slight increase in the number of PH3-positive cells in the Loa/Loa brain slices compared with wild type, this difference was not statistically significant (Figure 6A; *P *= 0.054; n = 5 brain slices per genotype). Similarly, counts of PH3-positive cells located in the ventricular or abventricular (>100 μm from the ventricle) regions were not significantly different between wild-type and Loa/Loa brains (Figure 6A; *P *= 0.068; n = 5 brain slices per genotype). As an additional test for impaired cell cycle progression, we performed a 2-hour BrdU pulse in E16 mice and found a similar number of BrdU-positive cells in wild-type versus mutant brain (Figure 6B). We also observed a comparable fraction of Pax6-positive cells between wild-type and Loa/Loa brains at both E16 and E18 (data not shown). Wild-type and Loa/Loa brains are of comparable size and have a similar cortical thickness (Figure 1); thus, together, these results argue against the gross mitotic defects that have been reported for a *NudE *null mouse [6].

### Axon extension is reduced in Loa/Loa neurons

**Figure 7:**
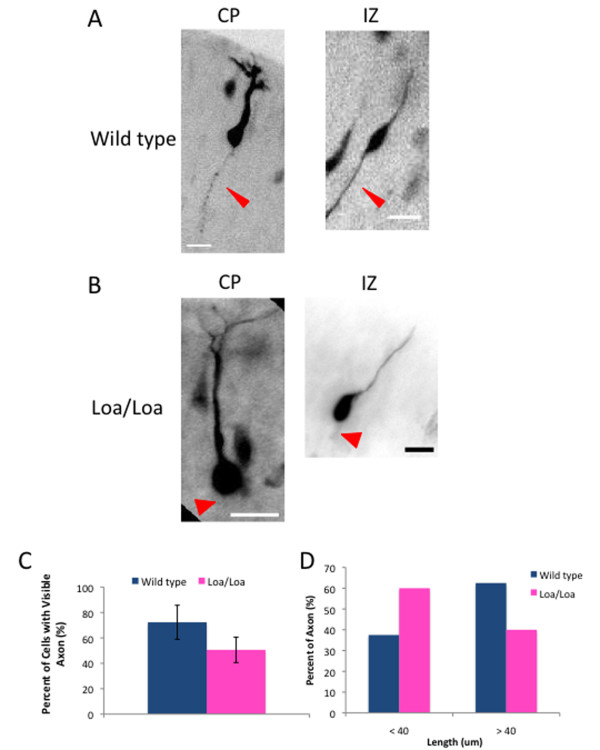
**Loa/Loa neurons display a defect in axon extension *in vivo***. **(A,B) **Individual neurons from Oregon Green stained wild-type (A) or Loa/Loa (B) brain sections with a visible axon (thin arrowhead), or no visible axon (wide arrowhead). Neurons from both the cortical plate (CP) and intermediate zone (IZ) were analyzed. Scale bars = 20 μm. **(C) **Quantification of the percentage of wild-type or Loa/Loa neurons with a visible axon. Only neurons with visible leading processes were scored for an axon, or lack thereof. Error bars represent the standard deviation. **(D) **Quantification of the percentage of wild-type and Loa/Loa axons that were shorter or longer than 40 μm.

**Figure 8:**
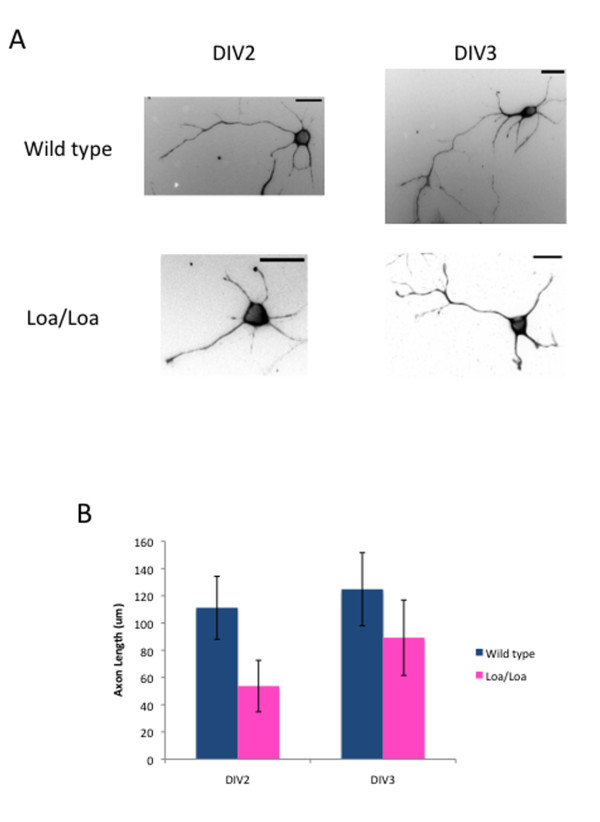
**Loa/Loa neurons exhibit a defect in axon extension *in vitro***. **(A) **Neurons were cultured from wild-type and Loa/Loa brains and grown for 2 or 3 days *in vitro *(DIV). Scale bars = 20 μm. **(B) **Graph displaying the average length of an axon (± standard deviation) from wild-type or Loa/Loa DIV2 or DIV3 neurons. An axon is defined here as one process that is at least 40 μm long and twice as long as the other processes.

Dynein and its regulatory factors, dynactin and LIS1, have been implicated in axon elongation in rat brain slices subjected to LIS1 RNAi and in cultured neurons [7,35,36]. Oregon Green BAPTA staining could detect clear trailing axons in a majority of radially migrating neurons in wild-type mice (Figure 7A,C; 72.3%; n = 32 cells from three brain slices); however, axons were detectable in fewer neurons in Loa/Loa slices (Figure 7B,C; 50.6%; n = 30 cells from three brain slices; *P *< 0.0001). Of the visible axons, 62.5% of the wild-type axons could be followed for longer than 40 μm compared with only 40.0% of the Loa/Loa axons (Figure 7D). We also observed a decrease in axon extension for Loa/Loa neurons *in vitro*. We cultured neurons on laminin for 2 or 3 days *in vitro *(DIV), then examined axonal length. At DIV2, the average axon length of wild-type neurons was 111 ± 23 μm compared with 54 ± 19 μm for the Loa/Loa neurons (n = 57 and 53 neurons, respectively; *P *< 0.0001), and by DIV3, the average axon length of wild-type neurons was 125 ± 27 μm compared with 89 ± 28 μm for the Loa/Loa neurons (n = 60 and 55 neurons, respectively; *P *< 0.0001) (Figure 8A,B). Together with our earlier results [23], these results reveal that dynein processivity is necessary for axon elongation.

### Loa/Loa mutant dynein can still bind its regulatory proteins

**Figure 9:**
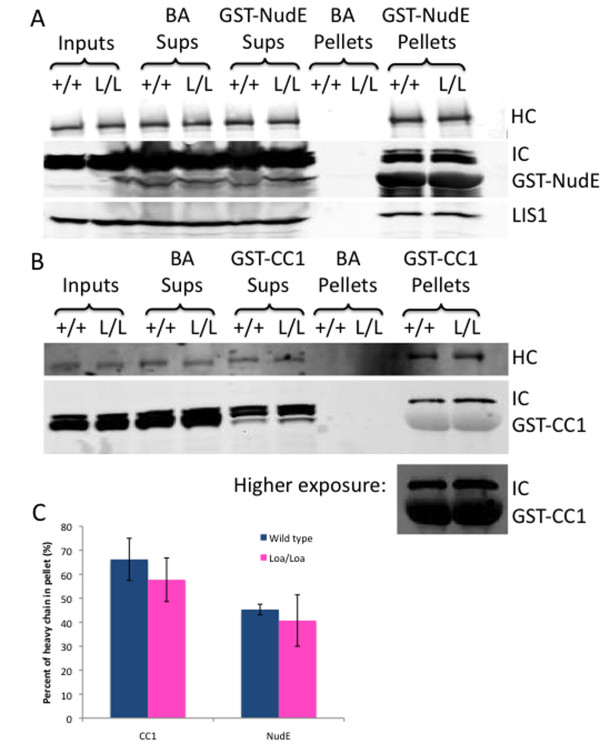
**Loa/Loa dynein can still bind its regulatory partners**. **(A) **GST-NudE pull-down assay from wild-type and Loa/Loa brain lysate. Input is 30% of total. Immunoblot reveals that protein is not pulled down by beads alone (BA), but similar amounts of both wild-type (+/+) and Loa/Loa (L/L) dynein (as shown by immunoblotting for dynein heavy chain and dynein intermediate chain) along with LIS1, are pulled down by GST-NudE. HC, heavy chain; IC, intermediate chain. **(B) **GST-CC1 pull-down assay from wild-type and Loa/Loa brain lysate. Input is 30% of total. Immunoblot reveals that dynein is not pulled down by beads alone (BA), but similar amounts of both wild-type (+/+) and Loa/Loa (L/L) dynein (as shown by immunoblotting for dynein heavy chain and dynein intermediate chain) are pulled down by GST-CC1. A higher exposure blot reveals the GST-CC1 band below the IC band. **(C) **Graph depicting the average percentage of wild-type or Loa/Loa dynein heavy chain (± standard deviation) that is pulled into the pellet by either GST-NudE or GST-CC1 (n = 3 experiments per genotype).

We previously reported that the Loa mutation specifically affects the processivity of dynein due to apparent miscoordination of the motor domains. We observed no change in the association of the mutant dynein with isolated neuronal membranes, further indicating that the physiological effects of Loa could be accounted for by altered mechanochemical activity alone [23]. However, because the cytoplasmic dynein regulators NudE, LIS1, and dynactin are also associated with brain development, we tested the mutant dynein further for its ability to interact with these factors. We performed pull-downs with purified proteins rather than immunoprecipitations with a dynein intermediate chain antibody, which might interfere with these interactions. Pull-downs from brain cytosol with recombinant glutathione S-transferase (GST NudE revealed no difference in the associated dynein or LIS1 between wild-type and Loa/Loa mutants (Figure 9A,C; 66.2 ± 8.8% and 57.7 ± 9.1% of heavy chain in the NudE pellet, respectively; *P *= 0.310). We similarly tested the GST-tagged CC1 fragment from the dynactin subunit p150^*Glued*^, which interacts with the dynein intermediate chain [37,38]. Comparable levels of wild-type and Loa/Loa dynein were again found in the CC1 pull-downs (Figure 9B,C; 45.3 ± 2.2% and 40.7 ± 10.7% of heavy chain in the pellet, respectively; *P *= 0.506). Therefore, the developmental defects we observe in the Loa/Loa mice likely reflect intrinsic abnormalities in the mutant dynein.

## Discussion

We find that Loa mutant mice exhibit defects in cortical and hippocampal development. Using live cell imaging techniques, we found direct evidence for a migration delay of bipolar neurons. Our results further indicate that this effect is not associated with defects in dynein interactions with LIS, NudE, or dynactin, and is therefore likely to result directly from the inherent mechanochemical defects caused by the Loa mutation [23].

The abnormal cortical lamination we observe in the Loa/Loa mouse brain is similar to that seen in LIS1 compound heterozygous mutant mice [5,9]. LIS1 mice exhibit severe disorganization of pyramidal cells in the CA1, CA2, and CA3 regions, as well as a reduced density of granule cells in the dentate gyrus [9,39]. We observe a clear delay in the migration of granule neurons in the Loa/Loa mouse to form the dentate gyrus. Although the formation of the dentate gyrus has not been examined in the LIS1 mice, a delay in granule cell migration could provide a potential explanation for the diminished size of the dentate gyrus in these mice.

We detected a decrease in axon extension in Loa/Loa neurons both *in vivo *and *in vitro*, as well as a decrease in axon length. Dynein as well as LIS1 are necessary for the reorganization and extension of the growth cone during axonogenesis [7,35,36]. These proteins are required to help microtubules extending into the peripheral zone of the growth cone resist retrograde actin flow [36]. A Loa mutant dynein detaches from microtubules about twice as frequently as the wild-type motor protein [23]. Therefore, microtubule penetration into the peripheral zone could be compromised, providing an explanation for altered axon elongation.

Altered expression of LIS, cytoplasmic dynein, and NudE have each been found to affect mitotic index in the developing brain [6,7,40]. These effects reflect direct roles for these proteins in mitosis [31,41,42]. Furthermore, LIS1 and cytoplasmic dynein RNAi interfere with apical migration of radial glial nuclei to the ventricular surface, which has been found to be essential for mitotic entry [7,43]. The similarity in mitotic index between Loa/Loa and wild-type mouse brains suggests that any effect on mitotic entry or progression must be relatively minor, though further live analysis of mitosis might still reveal altered mitotic behavior.

The current study indicates that the Loa mutation affects neuronal migration in a similar manner as reduced LIS1 expression [5,7,9]. However, the Loa mutation affects dynein processivity [23], whereas LIS1 contributes to dynein force production [44]. Our results indicate, therefore, that different defects in dynein function can lead to a common developmental outcome. The mechanical basis for this result is uncertain, but is likely a consequence of the coordinate behavior of multiple dynein molecules during high load functions. In *in vitro *studies, LIS1 was found to convert cytoplasmic dynein to a persistent force-producing state, though the level of force generation by individual dynein molecules was unaffected. However, force generation by multiple motors is summated, and this effect was further enhanced by LIS1 as revealed in *in vitro *laser trap bead assays and in computational simulations [44]. Thus, LIS1 appears to be ideally suited for a role in high load functions such as nuclear transport [12,44]. The Loa mutation, in contrast, had a clear effect on motor processivity [23]. How the Loa mutation might affect transport of high load structures is uncertain, but could also involve coordination of multiple dynein molecules. The Loa mutation results not only in decreased single molecule processivity *in vitro*, but also in decreased run-lengths for lysosomes/late endosomes *in vivo*, which are thought to be driven by an average of approximately five to seven dyneins [23,45,46]. Computational modeling indicates that, although cargo run length increases with motor number, the Loa processivity defect extends to the multimotor condition [23]. Thus, defects in neuronal migration could conceivably be explained by a reduction in run length during nuclear transport. We reason, however, that Loa should also affect aggregate force production by multiple motors, which is a reflection of the number of individual motors simultaneously 'engaged' with microtubules at any given time. This value should be reduced for Loa as a simple consequence of its increased microtubule detachment rate. Thus, the defect in neuronal migration we observe in the Loa/Loa mouse could result from shorter nuclear run lengths, decreased force, or both. The prolonged state of somal distortion we observe during neuronal migration in the Loa/Loa neurons could be indicative, in particular, of a reduction in nuclear translocation forces, but further work will be needed to directly determine the effects of the Loa mutation at the multiple motor level.

The defects we observe in the mutant neurons may explain the perilethality of the Loa/Loa mice. The Loa homozygotes die within 24 hours of birth due to an inability to move their mouths, and therefore feed. A 2-day delay in the migration of facial motor neurons to their destination may be responsible for this facial paralysis. Correspondingly, a delay in axon extension could also contribute to facial paralysis due to a lack of muscle innervation.

## Conclusions

Our results reveal a clear effect of the Loa mutation on neuronal migration, in addition to its previously reported effects on retrograde vesicular transport and on motor and sensory neuron viability. Our observations establish the utility of the Loa mouse as a hypomorphic dynein mutant model for the investigation of not only neurodegeneration but also general cytoplasmic dynein function.

## Materials and methods

### Animals

Mice were housed in standard cages on a 12:12 light:dark schedule at 25°C. All experimental research was performed according to the guidelines of the Institutional Animal Care and Use Committee (IACUC). All experimental analyses were performed on mutant animals with littermate-matched controls.

### Immunohistochemistry

Brains were fixed in 4% paraformaldehyde for 24 hours after extraction, then transferred to phosphate-buffered saline (PBS) until sectioning. Brains were sectioned on a Vibrotome (Leica VT 1200S) into 50-μm thick slices. For staining with primary antibodies, slices were blocked at room temperature for 1 hour in PBS with 10% donkey serum and 1% Triton X-100. For antibodies requiring antigen retrieval (such as Foxp2), slices were boiled in 10 mM sodium citrate buffer (pH 6), then cooled back to room temperature before blocking. Primary antibodies were applied overnight at 4°C in PBS with 1% donkey serum and 0.1% Triton-X-100 at the following dilutions: anti-ctip2, 1:500 (rat; Abcam, Cambridge, MA, USA); anti-foxp2, 1:200 (rabbit; Abcam); PH3, 1:500 (rabbit; Millipore, Billerica, MA, USA); Prox1, 1:500 (rabbit; Abcam); Pax6, 1:200 (rabbit; Covance, Princeton, NJ, USA); and TujI, 1:500 (mouse; Covance). Slices were then washed in PBS and incubated with Cy2, Cy3, or Cy5 secondary antibodies (1:1,000; Jackson ImmunoResearch Laboratories, West Grove, PA, USA). Sections were mounted onto slides using VECTASHIELD HardSet Mounting Medium with DAPI (VectorLabs, Burlingame, CA, USA), then imaged on an Olympus IX81 inverted microscope. At least three brains per genotype were analyzed for each experiment.

### BrdU labeling and analysis

For the BrdU birthdating assay in Figure 2, pregnant mice were injected intraperitoneally with BrdU at 100 μg/g of body weight at either E16 or E18. The BrdU-labeled females were left to give birth and their offspring were sacrificed on P0. BrdU-positive cells in the cerebral cortex were detected by immunostaining with an anti-BrdU antibody (1:200, rat; Abcam). This antibody required pre-treatment with 2N HCl for 15 minutes at 37°C prior to blocking. Staining was performed as described above. To analyze the BrdU-positive neurons in Loa/Loa mutants and their wild-type littermates, each brain section was divided into six equal regions, and the relative density and distribution of the BrdU-stained neurons in each region was plotted.

For the BrdU pulse-chase experiment in Figure 5B, pregnant mice were injected intraperitoneally with BrdU at 100 μg/g of body weight at E16. The mice were then sacrificed 2 hours after the injection to analyze the number of BrdU pulse-labeled cells at the ventricular surface in Loa/Loa and wild-type embryonic brains.

### *In utero *electroporation

Plasmids were transfected using intraventricular injection followed by *in utero *electroporation as previously described [25]. Briefly, 1 to 2 μl of GFP cDNA (concentration of 1 to 3 μg/μl) were injected into the ventricle of embryonic brains at E15. A pair of copper alloy oval plates attached to the electroporation generator (Harvard Apparatus, Holliston, MA, USA) transmitted five electric pulses at 40 V for 50 milliseconds at 1 second intervals through the uterine wall. The embryos were then sacrificed 3, 4, or 5 days later and their brains were prepared as described above. The number of GFP-labeled neurons at the ventricular zone, subventricular/intermediate zone, and cortical plate was plotted for Loa/Loa and wild-type littermates to assess the progression of neuronal migration from the ventricular surface.

### Organotypic slice cultures and live cell imaging

These experiments were performed using a modified procedure of previously described methods [28,47]. Briefly, E16 brains were collected and sectioned coronally on a vibratome (Leica), then placed in a 50-mm MatTek dish (MatTek Corporation, Ashland, MA, USA) containing imaging medium (F12/MEM medium with 10% fetal bovine serum and 1 × penicillin/streptomycin (Invitrogen, Carlsbad, CA, USA) with 10 μg/ml of Oregon Green BAPTA-1 488 AM (Invitrogen). Slices were then incubated for 1 hour at 37°C with 5% CO_2_. After the incubation, the slices were washed in warmed PBS, then covered with a thin layer of Matrigel (BD Biosciences, San Diego, CA, USA) diluted 70 × in imaging medium, then incubated again for 30 minutes. The dish was then imaged in 1 ml of additional imaging media with 10 mM Hepes at 37°C with 5% CO_2 _on an Olympus IX81 inverted microscope. Images were captured every 10 minutes for 3 to 5 hours using an Hamamatsu ORCA-R2 CCD camera and Metamorph software (Molecular Devices, Sunnyvale, CA, USA. The speed of migration and length of axon were analyzed with Metamorph software. The position of the soma center was measured in each image and displacement of the soma was defined by the difference in location of the center between consecutive frames. Therefore, the somal speed is described here as the displacement per unit time. The length of the axon was defined as the distance from the basal end of the soma to the tip of the process. Live cell imaging experiments and axon length measurements were performed on three brains per genotype.

### Biochemical assays

Whole brain lysates from P0 wild-type and Loa/Loa mouse pups were used for the NudE and CC1 pull-downs. For each experiment, the lysate was first pre-cleared with glutathione agarose 4B (USB Corporation, Cleveland, OH, USA) in order to prevent non-specific binding in the real assay. The beads were then spun down, and the pre-cleared lysate was used for the pull-downs. The pull-down solution contained the brain lysate, phosphate-glutamate buffer (10 mM sodium phosphate, 100 mM sodium glutamate, pH 7.0 with 1 mM MgSO_4_, 1 mM EDTA, 1 mM DTT, and 0.2% NP-40) up to 350 μl and either beads alone, 1 μg/μl GST-NudE [44] or 1 μg/μl GST-CC1 [37,38]. The solutions rotated at 4°C for 2 hours, then the beads were spun down to separate the supernatants and pellets. The supernatants and pellets from each pull-down (beads alone, NudE, or CC1) were collected and analyzed by SDS-PAGE and western blot probing for dynein heavy chain (rabbit, 1:500) [48] and dynein intermediate chain (mouse, 1:1500; clone 74.1 provided by K Pfister, University of Virginia, Charlottesville, VA, USA). In the case of the NudE pull-down, we also probed for LIS1 (mouse, 1:1500; clone 338, Sigma, St. Louis, MO, USA).

## Abbreviations

BrdU: 5-bromo-2'-deoxyuridine; CP: cortical plate; DIV: days *in vitro*; E: embryonic day; GFP: green fluorescent protein; GST: glutathione S-transferase; IZ: intermediate zone; Loa: Legs at odd angles; P: postnatal day; PBS: phosphate-buffered saline; PH3: phospho-histone 3; RNAi: RNA interference.

## Competing interests

The authors declare that they have no competing interests.

## Authors' contributions

KMOM and RBV designed experiments, KMOM performed experiments and analyzed data, and KMOM and RBV wrote the manuscript. Both authors read and approved the final manuscript.
